# Cued Fear Conditioning in Carioca High- and Low-Conditioned Freezing Rats

**DOI:** 10.3389/fnbeh.2019.00285

**Published:** 2020-01-24

**Authors:** Carolina Macêdo-Souza, Silvia S. Maisonnette, Claudio C. Filgueiras, J. Landeira-Fernandez, Thomas E. Krahe

**Affiliations:** ^1^Laboratório de Neurociência do Comportamento, Departamento de Psicologia, Pontifícia Universidade Católica do Rio de Janeiro, Rio de Janeiro, Brazil; ^2^Laboratório de Neurofisiologia, Departamento de Ciências Fisiológicas, Instituto de Biologia Roberto Alcantara Gomes, Centro Biomédico, Universidade do Estado do Rio de Janeiro, Rio de Janeiro, Brazil

**Keywords:** anxiety disorders, aversive learning, animal models of anxiety, cued fear, GAD, carioca high and low conditioned freezing lines

## Abstract

Anxiety disorders (AD) comprise a broad range of psychiatric conditions, including general anxiety (GAD) and specific phobias. For the last decades, the use of animal models of anxiety has offered important insights into the understanding of the association between these psychopathologies. Here, we investigate whether Carioca high- and low-conditioned freezing rats (CHF and CLF, respectively), a GAD animal model of anxiety, show similar high- and low-freezing behavioral phenotypes for cued auditory fear conditioning. Adult CHF (*n* = 16), CLF (*n* = 16) and normal age-matched Wistar rats (control, CTL, *n* = 16) were tested in a classical auditory-cued fear conditioning paradigm over 3 days (Tone + Shock and Tone only groups, *n* = 8 per treatment). Freezing responses were measured and used as evidence of fear conditioning. Overall, both CHF and CLF rats, as well as CTL animals displayed fear conditioning to the auditory CS. However, CLF animals showed a rapid extinction to the auditory conditioned stimulus compared to CHF and CTL rats. We discuss these findings in the context of the behavioral and neuronal differences observed in rodent lines of high and low anxiety traits.

## Introduction

Anxiety disorders (AD) are among the most prevalent and debilitating psychiatric conditions (Baxter et al., 2013), afflicting more than 250 million people worldwide (World Health Organization, 2017). AD poses significant economic, social, and emotional problems as it leads to reduced productivity, poor academic performance, increased health care treatment, stress, and family-based difficulties (Tyrer and Baldwin, 2006). Current estimates of the financial burden to society related to AD exceed $3 billion dollars per year (Chisholm et al., 2016). Moreover, longitudinal data indicate that AD is linked to a greater chance of developing depression, which in turn is associated with an increased risk of death by suicide (Sareen et al., 2005; Simon, 2009; Meier et al., 2016). Generalized anxiety disorder (GAD; Tyrer and Baldwin, 2006) and specific phobia (Eaton et al., 2018) are two of the most common AD (Wolitzky-Taylor et al., 2010; Steel et al., 2014). The former is characterized primarily by a persistent and uncontrollable state of wariness, whereas the latter is marked by apprehension and persistent fear towards specific objects or situations (American Psychiatric Association, 2013). While epidemiological studies provide ample evidence of comorbidity among different types of AD (Michael and Margraf, 2004; Bandelow and Michaelis, 2015), the interaction between GAD and specific phobia is not very clear (Lang et al., 2000; Grillon, 2002; Watson, 2005). Understanding the relationship between GAD and specific phobia at the neural and behavioral levels can provide further insight into the neurobiological underpinnings of these disorders.

Bidirectional selection of rodents for anxiety traits has been successfully used to investigate the basic pathophysiological mechanisms involved in AD (Lang et al., 2000; Willner and Mitchell, 2002; Belzung and Lemoine, 2011). Up to now, more than a handful of rat lines selectively bred for high and low anxiety-related behavior have been created (Gomes et al., 2013). Among such lines are the Roman High and Low Avoidance rats, the High and Low Anxiety-related Behavior rats (HAB and LAB, respectively), and the Carioca High- and Low-Conditioned Freezing rats (CHF and CLF), to name a few (Liebsch et al., 1998; Río-Álamos et al., 2017). Interestingly, although the high and low anxiety-traits of each one of these lines was based on a specific behavioral response (i.e., learning in a two-way avoidance task, time spent in the open arm of the elevated plus-maze, and conditioned freezing to contextual cues associated with foot shocks), rats from different lines frequently display similar high and low anxiety behavioral phenotypes when tested in other aversive learning and conditioning paradigms (Gomes et al., 2013). For instance, comparable behavioral differences between HAB and LAB rats were observed in social interaction, the open-field, and the light-dark box tests (Gomes et al., 2013). However, this consistency across measures is not observed amidst all lines (Gomes et al., 2013), indicating possible unique trait and state interactions. Particularly, when it comes to studies comparing conditioned anxiety responses to contextual cues (GAD model) and cue-specific stimuli (as in specific phobia), results have been, thus far, inconclusive (Lang et al., 2000; Curzon et al., 2009; Tovote et al., 2015). One possible reason for this inconsistency is that the animal lines used in previous studies were neither selectively bred based on behavioral responses to contextual, nor to specific cues. Thus, the aim of the present study was to evaluate the cued stimulus-conditioned freezing of the CHF and CLF rats, respectively, rat lines selectively bred for high and low freezing responses to contextual cues previously associated with an aversive stimulus (Gomes et al., 2011).

## Materials and Methods

In this study were used adult CHF (*n* = 16) and CLF (*n* = 16) male rats (13–17 weeks of age) obtained from the F28 outbreeding generation of the CHF and CLF lines, as described previously (de Castro Gomes and Landeira-Fernandez, 2008). Age-matched Wistar rats, composed of the offspring of randomized cross-breeding populations, thus encompassing animals with high, low and average conditioned freezing responses, were used as controls (CTL, *n* = 16). In order to control for litter effects, rats were randomly selected from a minimum of five different litters per line as well as for animals in the control group. All animals were bred and maintained in the animal facility of the Laboratory of Behavioral Neuroscience (LANEC) of the Pontifical Catholic University of Rio de Janeiro and kept on a 12:12 h light/dark cycle (lights on: 7:00 h, lights off: 19:00 h) at controlled temperatures (24 ± 1°C), with free access to food and water. At weaning (postnatal day 21), rats of the same group were housed together (six animals per cage). Prior to the beginning of behavioral testing, the number of animals per cage was reduced to four and each rat was handled once a day (2 min) for 5 days. Behavioral testing was performed during the light period of the light-dark cycle and carried out on three consecutive days. All experimental procedures were performed in compliance with the Animal Care and Use Committee of the State University of Rio de Janeiro (CEUA 036/2013), in accordance with the declaration of Helsinki and with the Guide for the Care and Use of Laboratory Animals as adopted and promulgated by the National Institutes of Health.

We used different sets of chambers to study auditory fear conditioning (four chambers A and four B). Since the Carioca lines were selectively bred for high- and low-conditioned freezing responses to contextual cues (de Castro Gomes and Landeira-Fernandez, 2008), visual, tactile and olfactory cues between chambers A and B were different in order to reduce the context contribution to auditory fear conditioning (Jacobs et al., 2010). Chambers A consisted in plexiglass boxes (25 × 20 × 20 cm, Insight; Ribeirão Preto) with metal grid floors and illuminated by 25 W red lights, whereas chambers B were standard polycarbonate rat housing cages (18 × 31 × 38 cm), with smooth floors and illuminated by 25 W white lights. Both sets of chambers were located inside of sound-attenuating compartments and, before and after each use, chambers A were cleaned with an ammonia solution (5%) and chambers B were cleaned with an isopropyl alcohol solution (70%). Moreover, before testing animals for conditioned fear to tone, all experimental groups were habituated to the new chamber B context for 8 min 24 h after the auditory cue fear session (day 1). The conditional fear to tone testing session only took place on the next day, when animals were placed again in chamber B. Video cameras located inside the sound-attenuating compartments recorded the animals’ behavior continuously during all testing sessions. For a particular session, four animals were tested at the same time. A trained observer, blind to the experimental group of the animal, quantified freezing episodes within a span of 2 s during specific periods of each testing session. Freezing, a classic measure of fear (Fanselow, 1980), was defined as a complete absence of movements except those related to breathing. Computer triggered foot shocks (unconditioned stimuli, US) were delivered *via* an interface connecting a shock generator (Insight; Ribeirão Preto) to the metal grid inside the chambers. Pure tone (1,000 Hz, 67 dB) auditory stimuli (conditioned stimuli, CS) were delivered *via* a speaker (Coulbourn Instruments) located above each chamber (A and B). Auditory and foot shock stimuli were paired during acquisition sessions carried out in chambers A, whereas the same auditory stimulus was presented alone during the testing sessions using chambers B (see below).

Auditory fear conditioning was performed using a protocol adapted from Anagnostaras et al. (2010). Briefly, on the conditioning day (acquisition), CHF, CTL and CLF rats were evenly assigned to the following groups: CHF Tone + Shock (*n* = 8), CHF Tone only (*n* = 8), CTL Tone + Shock (*n* = 8), CTL Tone only (*n* = 8), CLF Tone + Shock (*n* = 8), and CLF Tone only (*n* = 8). Animals were individually placed into chambers A (four animals tested at once) and habituated for 3 min (baseline). The fear acquisition was next elicited in the Tone + Shock groups by administering three foot-shocks of 1 mA each and 1 s in duration while presenting a 30 s audible cue, a 1,000 Hz pure tone of 67 dB. This procedure was repeated three times with an inter-trial interval of 2 min. At the end of the last audible cue presentation, freezing was scored for a period of 5 min. Tone only groups went through the same protocol except that they did not receive foot shocks. Animals were then returned to their home cages and taken back to the animal facility.

As mentioned above, to reduce the context contribution to auditory fear conditioning, 24 h after the fear acquisition, all animals were individually placed in a new context (chamber B) for 8 min. Freezing was scored for a period of 5 min immediately after animals were placed in the chamber. By the end of the session, animals returned to their home cages and were transferred back to the animal facility. Twenty-four hours later, animals went through the exact same protocol except that this time by the end of a 3-min period of habituation (baseline) the same pure tone presented on day 1 (CS) was played again for 5 min. Freezing was scored for both the baseline and CS periods.

All statistical analyses were performed using IBM SPSS 23 software (SPSS Inc., Chicago, IL, USA). Comparisons were carried out using repeated-measures analysis of variance (rANOVA). To reduce the likelihood of type 1 statistical errors that might result from repeated testing, results were evaluated first by global rANOVA using *Lineage* (CHF, CTL and CLF) and *Treatment* (Tone only and Tone + Shock) as between-subjects factors and *Time interval* (minute-by-minute) and *Day* (day 1: context A, day 2 and Day 3: context B) as within-subject factors. Whenever significant effects of *Lineage*, *Treatment*, *Day* and *Time interval*, or interactions between these factors were detected, appropriate lower-order ANOVAs were performed, followed by Bonferroni’s *post hoc* tests. For simplicity, we reported results based only on the averaged univariate F tests. Whenever the sphericity assumption was violated, we used the Greenhouse-Geisser correction, which adjusts the degrees of freedom, in order to avoid Type I errors. For all statistical tests, significance was set at *P* < 0.05 (2-tailed).

Given that the Global rANOVA indicated a significant interaction between all factors (*Lineage* × *Treatment* × *Day* × *Time interval*: *F*_(11.8,248.9)_ = 2.6, *P* < 0.001), separate lower-order rANOVAS were carried out for: (i) each day, maintaining all other factors in the analysis; and (ii) each lineage (CHF, CTL and CLF) or treatment (Tone only and Tone + Shock). In addition, lower-order rANOVAs were performed across the different testing days comparing: (i) the first 3 min of all days (i.e., pre-CS of day 1 vs. comparable period on day 2 vs. pre-tone period of day 3); and (ii) the last 5 min (i.e., post-conditioning of day 1 vs. comparable period on day 2, and CS presentation of day 3).

## Results

In tone fear conditioning procedures, there is strong evidence that context plays a significant role in memory retrieval, by either acting as a competing cue or as a priming stimulus (Spear, 1973, 1978; Bouton, 1993; Denniston et al., 2001; Bouton et al., 2006; Stout and Miller, 2007; Urcelay and Miller, 2010). To minimize this confounding effect, many authors support the idea that conditioning and testing chambers should be as different as possible (Curzon et al., 2009; Anagnostaras et al., 2010; Jacobs et al., 2010), especially for animals predisposed to show high context generalization like CHF rats (Jacobs et al., 2010). To that end, in the Supplementary Material section, we provide additional evidence of the long-lasting effects of context fear conditioning in CHF and control animals (Supplementary Figures S1, S2; please see Supplementary Material for detailed methods and explanatory figures).

**Figure 1:**
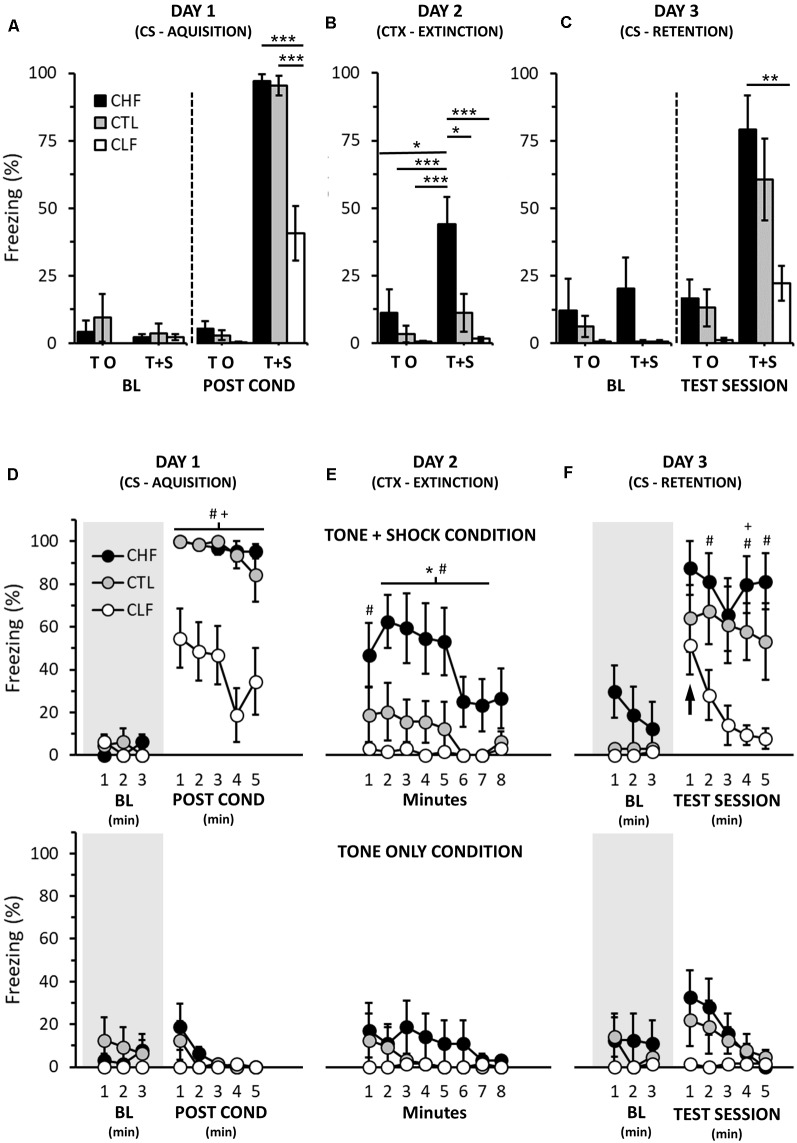
Cued fear conditioning in Carioca high-conditioned freezing (CHF), Carioca low-conditioned freezing (CLF), and control (CTL) animals. **(A–C)** Graphs showing the average freezing responses (in %) for each day. In **(D–F)**, freezing responses (also in %) are depicted across the same days and conditions along 1-min intervals (see “Materials and Methods” section). Also in **(D–F)**, top row panels illustrate responses of rats that on day 1 were exposed to both acoustic and foot shock stimuli (Tone + Shock groups), whereas bottom row panels depict responses of animals that on day 1 were only exposed to the acoustic stimulus (Tone only groups). Shaded areas portray baseline periods with no stimulus presentations (tone or tone accompanied by foot shocks). Note that no significant differences in freezing were found for all Tone + Shock group comparisons in the first minute of the CS retention trial (day 3, upward-pointing arrow, right top panel). BL, baseline; CS, conditioned stimuli; CTX, context. Bars and symbols are means ± SEM. In **(A–C)**, Bonferroni *post hoc* tests, *P < 0.05, ^**^P < 0.01, ^***^P < 0.001. In **(D–F)**, Bonferroni *post hoc* tests, CHF vs. CTL, *P < 0.05; CHF vs. CLF, ^#^*P* < 0.05; CTL vs. CLF, ^+^*P* < 0.05.

On day 1 of cue fear conditioning, there were no significant differences or interactions regarding the amount of freezing between all experimental groups prior to the presentation of the auditory and foot shock stimuli (Figures 1A,D; baseline; rANOVA, *Time Interval*: *F*_(1.3,55.7)_ = 0.7, *P* = 0.46; *Lineage*: *F*_(2,42)_ = 0.8, *P* = 0.44; *Treatment*: *F*_(1,42)_ = 0.3, *P* = 0.59; *Time Interval × Lineage × Treatment*: *F*_(2.6,55.7)_ = 0.8, *P* = 0.49). However, following CS acquisition, animals that received foot shocks paired with the auditory stimuli (Tone + Shock groups) displayed significant more freezing than those that did not receive foot shocks paired to auditory stimuli (Tone only groups, Figures 1A,D; rANOVA, *Treatment*: *F*_(1,42)_ = 379.5, *P* < 0.001). A separate rANOVA performed for Tone + Shock groups indicated that freezing responses after CS acquisition were significantly greater compared to their respective baseline values (Figures 1A,D; *Time Interval*: *F*_(2.3,57.5)_ = 114.7, *P* < 0.001). Moreover, CHF and CTL Tone + Shock rats exhibited significantly more freezing than CLF ones in the same experimental condition (Figures 1A,D; *Lineage*: *F*_(2,42)_ = 22.1, *P* < 0.001).

On day 2, freezing averages of Tone + Shock groups were significantly smaller than their respective values observed on the post-conditioning phase of day 1 (compare Figures 1A,B and Figures 1D,E; top panels; rANOVA, *Day*: *F*_(1.0,42.0)_ = 24.2, *P* < 0.001; *Day × Treatment*: *F*_(1.0,42.0)_ = 37.6, *P* < 0.001). Although smaller, Tone + Shock CHF animals still displayed significantly more freezing than CTL and CLF Tone + Shock and Tone only groups both for the total average and along day 2 session (Figures 1B,E; rANOVA, *Lineage × Treatment*: *F*_(2.0,42.0)_ = 3.5, *P* < 0.05). Total freezing averages of CTL and CLF Tone + Shock rats did not differ from their Tone only group counterparts (Figures 1B,E). Lastly, freezing values (overall, first 3, and last 5 min) of Tone only groups were not statistically different from their baseline values of day 1 (compare Figures 1A,B and Figures 1D,E; bottom panels; rANOVAs, first 3 min, *Day*: *F*_(1.0,21.0)_ = 1.9, *P* = 0.18; last 5 min, *Day*: *F*_(1.0,21.0)_ = 0.3, *P* = 0.86).

On day 3, the baseline freezing average of CHF Tone + Shock rats was significantly smaller compared to CHF Tone + Shock values of day 2 (compare Figures 1B,C; rANOVA: *Day*: *F*_(1.07.0)_ = 22.8, *P* < 0.01). Further, and perhaps more importantly, overall baseline values of CHF Tone + Shock and CHF Tone only groups were similar (Figures 1C,F; rANOVA, *Treatment*: *F*_(1,14)_ = 0.3, *P* = 0.62; *Time interval × Treatment*: *F*_(2,19)_ = 1.4, *P* = 0.26). Analogous results were observed when the same comparisons were made among CTL (Tone + Shock and Tone only) and CLF (Tone + Shock and Tone only) groups (Figures 1B,C,E,F; rANOVAs, CTL: *Treatment*: *F*_(1,14)_ = 0.8, *P* = 0.47; *Time interval × Treatment*: *F*_(2,19)_ = 1.8, *P* = 0.19; CLF: *Treatment*: *F*_(1,14)_ = 0.0, *P* = 1.00; *Time interval × Treatment*: *F*_(2,19)_ = 0.0, *P* = 1.00).

Regarding conditioning to the presentation of the auditory cue, all Tone + Shock groups exhibited significantly higher overall freezing values compared to both their respective baseline values and their Tone only counterparts (Figure 1C; rANOVA, *Treatment*: *F*_(1,42)_ = 32.7, *P* < 0.001). Moreover, while CHF and CTL Tone + Shock rats did not differ in the overall amount of freezing during the testing session of day 3, CHF animals exhibited significantly more freezing than CLF ones (Figure 1C; Tone + Shock groups, rANOVA, *Lineage*: *F*_(2,21)_ = 5.9, *P* < 0.01). Interestingly, however, such difference only became apparent as the session progressed (i.e., in the first minute, all Tone + Shock experimental groups showed similar freezing responses, Figure 1F; top panel).

**Figure 2:**
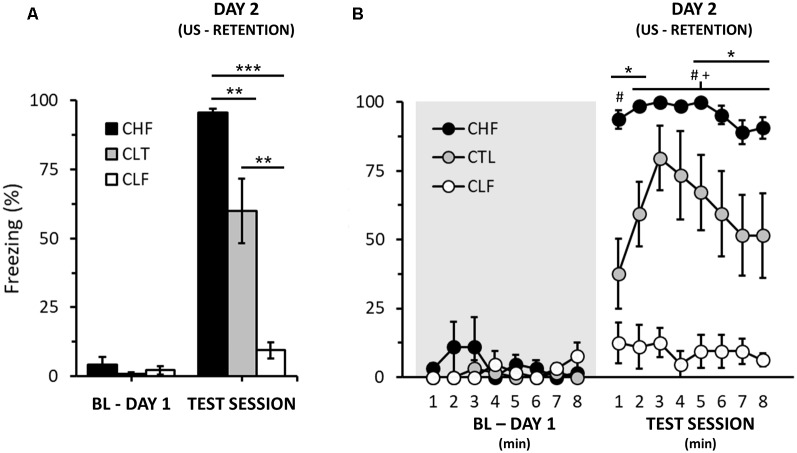
Effect of context on the freezing responses of CHF, CLF, and control (CTL) rats. **(A,B)** Graphs illustrating the average freezing responses (in %) of animal groups 24 h after context fear conditioning for the whole session **(A)** and within 1-min intervals **(B)**. The shaded area indicates baseline period with no foot shock stimulus presentation (day 1, see Supplementary Material). Bars and symbols are means ± SEM. In **(A)**, Bonferroni *post hoc* tests, ^**^P < 0.01, ^***^P < 0.001. In **(B)**, Bonferroni *post hoc* tests, CHF vs. CTL, *P < 0.05; CHF vs. CLF, ^#^*P* < 0.05; CTL vs. CLF, ^+^*P* < 0.05.

Finally, for the purposes of comparison, we also evaluated context fear conditioning in a separate sample of CHF, CTL and CLF animals (Figure 2; please see Supplementary Material for experimental details). Note that contrary to cued fear conditioning (Figure 1; day 3), CHF animals clearly displayed more freezing than both CTL and CLF rats (Figure 2; Day 2 only, rANOVA, *Lineage*: *F*_(2,21)_ = 38.7, *P* < 0.01). In addition, as expected, CLF animals exhibited very low levels of freezing to context fear conditioning both for the total average and along the testing session (Figure 2).

## Discussion

Here, we show that Carioca rats, as well as control animals, exhibited fear conditioning to an auditory CS. However, for the CLF group, such conditioning was rapidly extinguished. Nonetheless, although short-lived, the amount of freezing of the CLF Tone + Shock group to the auditory-conditioning stimulus was much greater than that displayed during the baseline period of the same day (Figure 1) or compared to the total freezing observed for context fear conditioning from a separated set of CLF animals (Figure 2).

The contextual and cue auditory fear conditioning findings presented here for CHF and CLF rats show different conditioning patterns. In the contextual fear, similar to previous observations from our group (Gomes et al., 2011; Hassan et al., 2013, 2015; León et al., 2017), CHF animals froze more than CTL rats and these more than CLF ones. In addition, CHF and CLF displayed very small within-group variability. On the other hand, in the cued auditory fear conditioning, no differences in the amount of freezing were observed between CHF and CTL groups during the auditory CS presentation and, for the first minute of the testing session, CLF rats also presented comparable freezing values. This pattern is similar to that seen in an earlier study using other lines of high and low anxiety rats. Muigg et al. (2008) showed that HAB/LAB rats behave equally with regard to cued auditory fear conditioning but exhibit striking differences (HAB > LAB) in the elevated plus-maze.

One possible explanation for the lack of consistency across measures (i.e., similar high and low anxiety behavioral phenotypes) between the two types of conditioning (context vs. cued fear) is that the neural pathways involved in these two types of the paradigm are distinct. Indeed, a substantial body of evidence from neurochemical, pharmacological, brain injury and electrical stimulation studies support this idea (for a review, see Tovote et al., 2015). For instance, the hippocampus plays a critical role in contextual fear conditioning and is not necessarily associated to cued fear acquisition (Phillips and LeDoux, 1992; Kim et al., 1993; Young et al., 1994; Corcoran and Maren, 2001; Sanders et al., 2003; Corcoran et al., 2005; Chaaya et al., 2018). On the other hand, the amygdala is essential for cued fear learning and may not be that important in contextual fear conditioning (Phillips and LeDoux, 1992; Vazdarjanova and McGaugh, 1998). In accordance to this dichotomy, previous neurochemical findings from our laboratory demonstrated that CHF and CLF rats differ in the amount of GABA content in the hippocampus (Dias et al., 2014), whereas electrolytic lesions of the amygdala lead to similar disruptions in conditioned freezing behavior in both CHF and CLF animals (de Castro Gomes and Landeira-Fernandez, 2008). Accordingly, unpublished immunocytochemistry data from our group using c-fos as a marker of neuronal activation indicates that CHF and CLF rats show similar levels of c-fos expression in the amygdala. Thus, our findings corroborate the assumption that conditioned anxiety responses to contextual and specific (auditory) cues encompass different anxiety traits and different neuronal substrates. In addition, our results suggest that specific neuronal changes are involved in the bidirectional selection of the Carioca lines for contextual fear.

In contrast to what we observed for CHF and CTL rats, CLF animals in the Tone + Shock group showed a significant reduction of the amount of freezing overtime during the presentation of the auditory cue (day 3). This result clearly points toward a faster extinction of the conditioned auditory fear in CLF rats and is in accordance with a previous study showing that LAB rats also display quick extinction to cued auditory fear conditioning (Muigg et al., 2008). A possible interpretation for these findings is that animals with a low anxiety trait are behaviorally more hyperactive than their counterparts with high anxiety, and thus quickly engage in active behaviors. It could be that CLF rats show excessive motor/action impulsivity, poor inhibitory control over previously conditioned, pre-potent responses (Cabib et al., 2002; Bari and Robbins, 2013). In a recent study, Yen et al. (2013) observed that mice selectively bred for low levels of anxiety showed both elevated levels of locomotion and deficits in habituation in the open field test in comparison to mice bred for normal and high anxiety-related behavior. Moreover, hyperactivity of low anxiety mice was attenuated by amphetamine treatment. Based on these results the authors propose the use of their low anxiety genetically selected mice as an animal model of attention deficit hyperactivity disorder (ADHD). In comparison, it is reasonable to think that the swift decline in the freezing behavior of CLF rats could be due to an analogous ADHD phenotype.

Another possibility is that CLF rats have a heightened threshold for pain. This, in turn, would translate into a faster extinction since there is a direct relationship between foot shock intensity and the strength of fear conditioning, the stronger the shock (US), the greater the amount of fear response (Fanselow, 1980). In other words, during acquisition (day 1) the experienced sensation of pain elicited in CLF rats is diminished due to an increased pain threshold, allowing for a faster extinction of the conditioned fear response. Note, however, that despite differences in pain threshold, CLF rats, like CHF and CTL animals, displayed a conditioned fear response to the auditory CS. A third, less likely possibility to explain the fast and progressive decrease of conditioned fear in CLF rats is that for these animals the inhibitory learning processes that allow for the extinction of conditioned fear responses occurs rather rapidly (Herry et al., 2010). In sum, further studies are needed to investigate whether one or more of these possibilities may explain the rapid extinction of conditioned auditory fear seen in CLF rats.

As mentioned in the results section, context plays a significant role in CS conditioning. Although space limitations prevent a thorough discussion of the topic, it deserves mention that Bouton (1988, 1993, 1994) has demonstrated that rats exhibit increased CS fear responses when the retention session takes place in a context other than the one in which acquisition occurred. While, we cannot discard that the change of context influenced CS fear recovery of CHF and CLF animals, we show that the experience that took place during day 2 (change of context) did not reinstate the learning that occurred during CS acquisition. On that note, the Carioca line could be an interesting model to further investigate CS-US context relationships, as on one hand, context exerts strong influence on CHF animals, and on the other hand, has little or no effect on CLF ones.

Past studies have characterized the Carioca line as a reliable rat model of innate anxiety (for a review, see Gomes et al., 2011). Apart from context fear conditioning, CHF rats display more anxious like behaviors than CLF rats in the elevated plus maze, less social interactions than CTL animals, and higher plasma corticosterone concentrations compared to both CLF and CTL animals (Dias et al., 2009; Salviano et al., 2014; Mousovich-Neto et al., 2015). Similar result patterns were not observed for behavioral tests measuring depression traits and cognitive skills. For instance, no differences between CHF and CLF rats were found in the forced swimming test, Morris water maze, and object recognition (Dias et al., 2009, 2014). These findings, together with our results demonstrating differences between cued and context fear conditioning in Carioca rats, reinforce the behavioral construct of this line as a rat model of GAD.

In comparison to other selectively bred lines for high and low levels of anxiety, our results are similar to previous ones in the sense that some lines show behavior discrepancies when tested in paradigms other than the one originally used for selective breeding (for a review, see Gomes et al., 2013). However, it is important to emphasize that CHF rats still presented stronger responses to CS conditioning than CLF animals. Likewise, Roman low avoidance rats (RLA/Verh) were found to display more freezing than Roman high avoidance ones (RHA/Verh) to contextual cues and to a CS previously associated with foot-shocks (Aguilar et al., 2002; López-Aumatell et al., 2009). Similar context conditioning results were observed between RLA and RHA inbred rats (Escorihuela et al., 1997). Thus, the nature of the threatening event seems to play an important role in the behavioral similarities and dissimilarities across measures among selectively bred lines for high and low levels of anxiety.

In conclusion, our findings show no overgeneralization of CHF fear responses to CS compared to CTL and CLF animals and are in accordance with previous preclinical and epidemiological studies (Lang et al., 2000; Grillon, 2002; Bandelow and Michaelis, 2015). This observation together with the systematic study of fear conditioning in normal and bidirectionally selected lines of high and low anxiety traits may shed light on the intricate relationship between the different types of AD.

## Data Availability Statement

The datasets generated for this study are available on request to the corresponding author.

## Ethics Statement

The animal study was reviewed and approved by Animal Care and Use Committee of the State University of Rio de Janeiro.

## Author Contributions

CM-S, SM and JL-F designed the research. CM-S and SM performed the research. CM-S, CF, TK and JL-F analyzed the data. CM-S and TK wrote the manuscript. SM, CF and JL-F contributed to the preparation of the manuscript. All authors revised and approved the final version of the manuscript.

## Conflict of Interest

The authors declare that the research was conducted in the absence of any commercial or financial relationships that could be construed as a potential conflict of interest.

## Supplementary Material

FIGURE S1 Effect of context on freezing responses of CHF and control (CTL) rats. **(A–B)** Graphs illustrating the average freezing responses (in %) of animals that, after 12 days, were re-exposed to chamber A (A × A condition) or exposed to chamber B (A × B condition). Freezing data is presented for the total **(A)** and within **(B)** the test session. Note that chambers A and B are the same ones used for CS conditioning experiments and that, also similar to the CS protocol, conditioning was always performed in chamber A (see “Materials and Methods” section). Bars and symbols are means ± SEM. In **(A)**, *Planned comparisons*: AxA vs. AxB, ^***^*P* < 0.001. In **(B)**, *Planned comparisons*: CHF: AxA vs. AxB, ^**^*P* < 0.01, ^***^*P* < 0.001; CTL: AxA vs. AxB, ^#^*P* < 0.05.

FIGURE S2 Context conditioning extinction over time in CTL animals. **(A,B)** Graphs illustrating the average freezing responses (in %) of animals for each **(A)** and across **(B)** test sessions. Bars and symbols are means ± SEM.
