# Supplementary figures and images for: Cued Fear Conditioning in Carioca High- and Low-Conditioned Freezing Rats
Source: Front Behav Neurosci. 2020 Jan 24;13:285. doi: 10.3389/fnbeh.2019.00285 (PMC6992609; doi:10.3389/fnbeh.2019.00285)

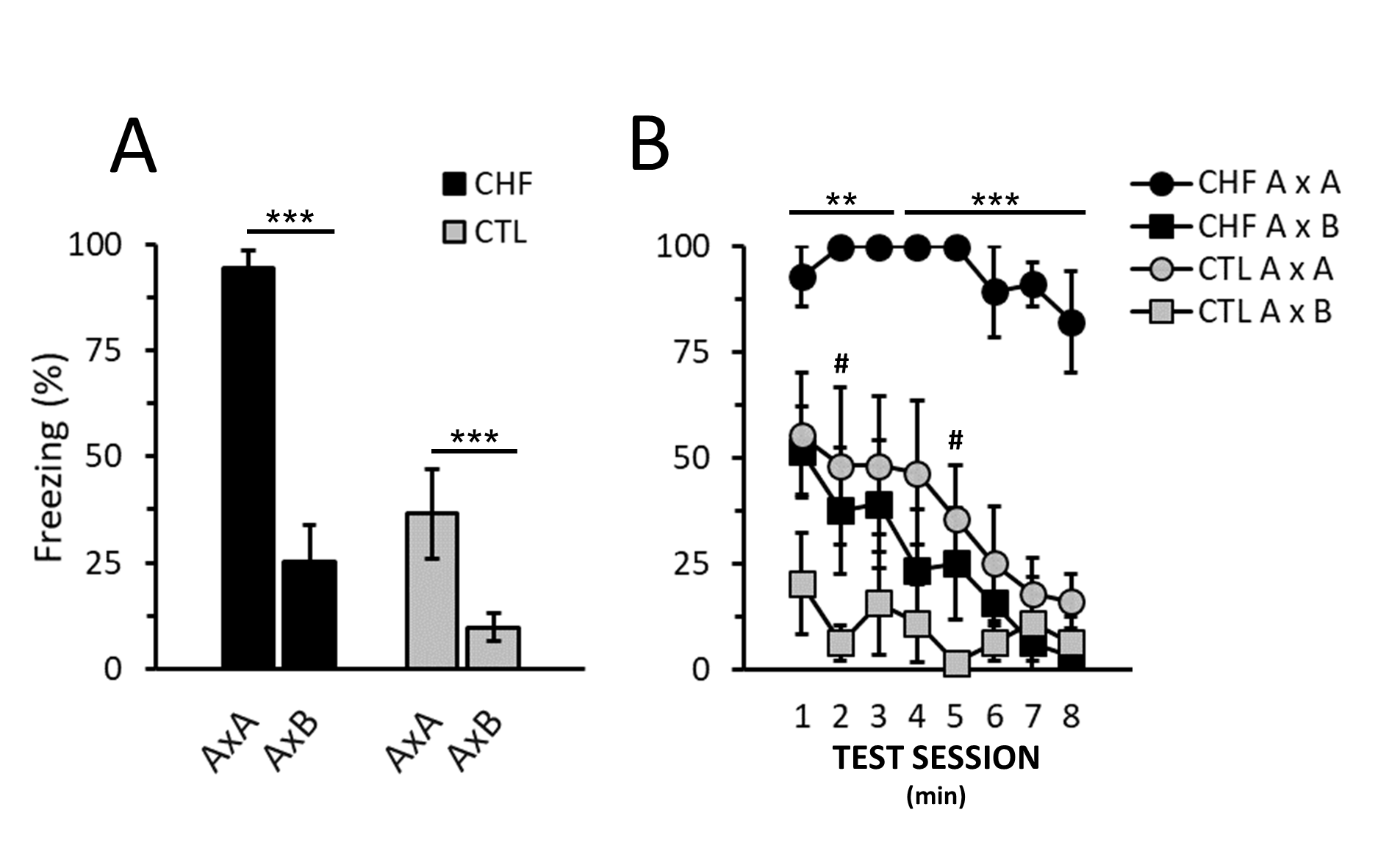

Supplement: FIGURE S1 — Effect of context on freezing responses of CHF and control (CTL) rats. (A–B) Graphs illustrating the average freezing responses (in %) of animals that, after 12 days, were re-exposed to chamber A (A × A condition) or exposed to chamber B (A × B condition). Freezing data is presented for the total (A) and within (B) the test session. Note that chambers A and B are the same ones used for CS conditioning experiments and that, also similar to the CS protocol, conditioning was always performed in chamber A (see “Materials and Methods” section). Bars and symbols are means ± SEM. In (A), Planned comparisons: AxA vs. AxB, ***P < 0.001. In (B), Planned comparisons: CHF: AxA vs. AxB, **P < 0.01, ***P < 0.001; CTL: AxA vs. AxB, #P < 0.05. [file Image_1.TIF]

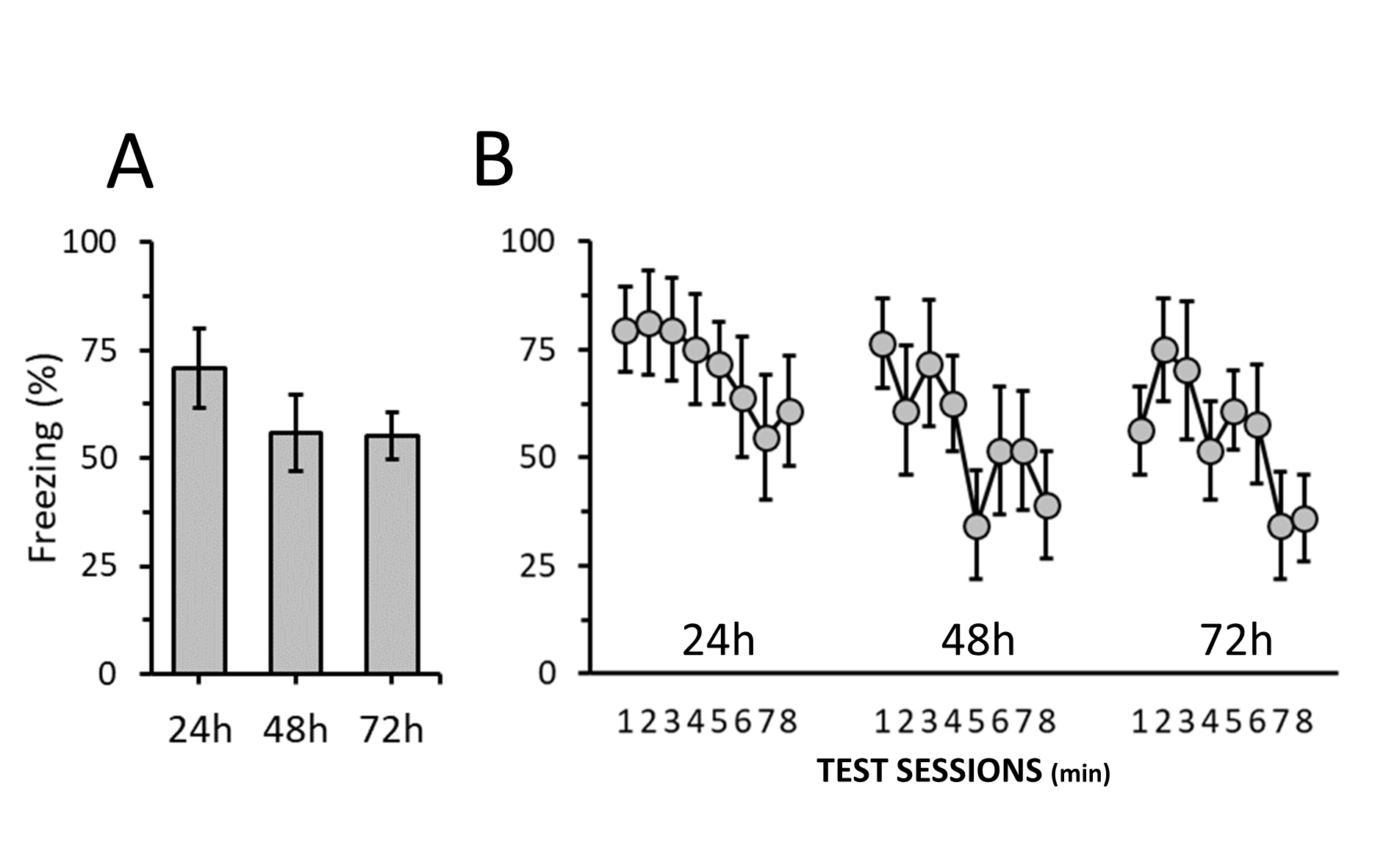

Supplement: FIGURE S2 — Context conditioning extinction over time in CTL animals. (A,B) Graphs illustrating the average freezing responses (in %) of animals for each (A) and across (B) test sessions. Bars and symbols are means ± SEM. [file Image_2.TIF]
